# Association between funding source, methodological quality and research outcomes in randomized controlled trials of synbiotics, probiotics and prebiotics added to infant formula: A Systematic Review

**DOI:** 10.1186/1471-2288-13-137

**Published:** 2013-11-13

**Authors:** Mary N Mugambi, Alfred Musekiwa, Martani Lombard, Taryn Young, Reneé Blaauw

**Affiliations:** 1 Division of Human Nutrition, Faculty of Medicine and Health Sciences, Stellenbosch University, P.O Box 19063, Tygerberg 7505, South Africa; 2 Centre for Evidence-Based Health Care, Faculty of Medicine and Health Sciences, Stellenbosch University, Stellenbosch, South Africa

**Keywords:** Synbiotics, Probiotics, Prebiotics, Funding source, Methodological quality

## Abstract

**Background:**

There is little or no information available on the impact of funding by the food industry on trial outcomes and methodological quality of synbiotics, probiotics and prebiotics research in infants. The objective of this study was to compare the methodological quality, outcomes of food industry sponsored trials versus non industry sponsored trials, with regards to supplementation of synbiotics, probiotics and prebiotics in infant formula.

**Methods:**

A comprehensive search was conducted to identify published and unpublished randomized clinical trials (RCTs). Cochrane methodology was used to assess the risk of bias of included RCTs in the following domains: 1) sequence generation; 2) allocation concealment; 3) blinding; 4) incomplete outcome data; 5) selective outcome reporting; and 6) other bias. Clinical outcomes and authors’ conclusions were reported in frequencies and percentages. The association between source of funding, risk of bias, clinical outcomes and conclusions were assessed using Pearson’s Chi-square test and the Fisher’s exact test. A p-value < 0.05 was statistically significant.

**Results:**

Sixty seven completed and 3 on-going RCTs were included. Forty (59.7%) were funded by food industry, 11 (16.4%) by non-industry entities and 16 (23.9%) did not specify source of funding. Several risk of bias domains, especially sequence generation, allocation concealment and blinding, were not adequately reported. There was no significant association between the source of funding and sequence generation, allocation concealment, blinding and selective reporting, majority of reported clinical outcomes or authors’ conclusions. On the other hand, source of funding was significantly associated with the domains of incomplete outcome data, free of other bias domains as well as reported antibiotic use and conclusions on weight gain.

**Conclusion:**

In RCTs on infants fed infant formula containing probiotics, prebiotics or synbiotics, the source of funding did not influence the majority of outcomes in favour of the sponsors’ products. More non-industry funded research is needed to further assess the impact of funding on methodological quality, reported clinical outcomes and authors’ conclusions.

## Background

There are numerous studies that explore the relationship between industrial sponsorship of biomedical research and published outcomes [1]. Several reviews have documented how trials funded by industry are more likely to report results in favour of the sponsor’s products [2-5]. These reviews focused on trials sponsored by the pharmaceutical industry. Few reviews have explored the impact of funding by the food industry on outcomes of research trials [6,7]. A review by Nkansah et al. also found that majority of trials investigating the effects of calcium supplementation in healthy children were industry funded and all supported calcium supplementation, in favour of the sponsor [8]. Similarly, a review by Lesser et al. found that scientific nutrition related articles (intervention trials, observational studies and scientific reviews) on common consumed beverages (soft drinks, juice, milk) funded by the food industry, were more likely to be favourable to the sponsor than articles that did not have industry funding [6].

Reporting only positive outcomes in a research trial significantly reduces a sponsors’ financial risk. Pressure to show a food product causes favourable outcomes in a specific population, may result in biases in trial design (methodology) and reporting of outcomes in industry sponsored research. This type of bias in nutrition research could adversely affect public health. Results from nutrition research also influence policy formulation, professional dietary guidelines, design of public health interventions and regulation of food product health claims. In addition, findings from nutrition research often receive publicity from the media, which influences consumer behaviour [6].

More studies are needed to explore the relationship between the food industry and nutrition research [7]. There is little or no information available on the impact of funding by the food industry on trial outcomes and methodological quality of synbiotics, probiotics and prebiotics research in infants. There are no systematic reviews that have explored if sources of funding affects outcomes and methodological quality of randomized controlled trials (RCTs) conducted on infants given probiotics, prebiotics or synbiotics supplemented infant formula.

Probiotics are defined as “live microorganisms” which when administered in adequate amounts may confer a health benefit to the host [9]. The main probiotic organisms that are currently used worldwide belong to the genera Lactobacillus and Bifidobacteria and are found in the gastrointestinal microflora [9]. The probiotics preparations of interest for this review are those added to infant formulas. Prebiotics are non- digestible food ingredients that may benefit the host by selectively stimulating the growth and/or activity of one or a limited number of bacteria in the colon and improving the host’s health [10-12]. The most widely studied prebiotics are galactooligosaccharides (GOS), inulin and fructooligosaccharide (FOS) [13,14]. GOS, FOS and inulin are added to different foods as fat and sugar replacements to improve texture or for their functional benefits [10,15,16]. When probiotics and prebiotics are administered simultaneously, the combination is termed Synbiotics.

The aim of this review was to explore whether financial sponsorship by the food industry affects outcomes and methodological quality of trials on synbiotics, probiotics or prebiotics used in infants. Methodological quality may be compromised when insufficient information is provided regarding sequence generation, allocation concealment, blinding, bias introduced from other sources and incomplete outcome reporting.

### Objective

The objective of this systematic review was to compare the methodological quality and outcomes of food industry sponsored trials versus non industry sponsored trials with regards to supplementation of synbiotics, probiotics and prebiotics in infant formula.

### Hypothesis

The source of funding in research trials using probiotics, prebiotics or synbiotics supplemented formula in infants is associated with outcomes in favour of the sponsor’s products and authors’ conclusions.

## Methods

### Criteria for considering studies for this review

#### Types of studies

All randomized controlled trials (RCTs) conducted from 1980 to 2012 (irrespective of language) on synbiotics, probiotics, or prebiotics added to infant formula were included. Study participants were healthy full term infants (>37 weeks gestation or > 2.5 kg birth weight, 0–12 months old), preterm infants (born < 37 weeks gestation), low birth weight (<2.5 kg at birth) and extreme low birth weight infants (<1000 g at birth). Infants were fed either infant formula (preterm or full term formula), mixed feeds (breast milk with infant formula) with added synbiotics, probiotics or prebiotics or conventional infant formula with or without placebo. RCTs were excluded if they included infants with cardiac defects, pulmonary diseases, gastrointestinal diseases, major congenital abnormalities or chromosomal abnormalities. Commentaries, editorials, letters to the editor and studies that were not RCTs were excluded.

#### Types of outcome

The outcomes included: 1) Source of funding, 2) Methodological quality (Risk of bias), 3) Clinical outcomes in RCTs, 4) Conclusions (Overall study conclusions and conclusions on reported clinical outcomes) and 5) Association between source of funding and methodological quality, clinical outcomes and author’s conclusions.

### Search methods for identification of studies

A literature search regardless of language was conducted on electronic databases including The Cochrane CENTRAL Register for Controlled Trials (2012), EMBASE (1980+), Scopus (1980 to 2012), EBSCO host (1960 to 2012), PUBMED / MEDLINE (1966 to 2012), OVID (1950 to 2012), SPORTDiscus (1960 to 2012), Web of Science (1970 to 2012), Science Direct (1950 to 2012), CINAHL (1980 to 2012), Science citation index (1970 to 2012), Latin American Caribbean Health Sciences literature (LILACS) (1965 to 2012), NLMGateway (1950–1966). RCTs published in non-English language journals were translated by independent translators who were familiar with the subject matter.

The search strategy used to search PUBMED for studies on full term infants is: (synbiotic* and probiotic* OR prebiotic*) AND (FOS or fructooligosaccharide or inulin or GOS or galactooligosaccharide) AND (infant formula* OR infant feeding OR formula OR formula milk) AND (infant* or baby or babies) NOT (preterm or premature or low birth weight babies or allergy or eczema) AND (randomized controlled trial* OR controlled clinical trial* OR random allocation*) Limits: Humans. This search strategy was modified to search other electronic databases and for studies on preterm infants.

A hand search was conducted on abstracts of major conference proceedings such as the Pediatric Academic Society meetings from 1990 (http://www.pas-meetings.org), cross checked references cited in RCTs and in recent reviews (published from 2003 to 2012) for additional RCTs not identified by electronic searches and speciality journals which were not included in any database such as Pediatrika and Chinese Journal of Microecology. To identify on-going and unpublished trials, experts in the field, manufacturers of infant formula containing probiotics and prebiotics were contacted. Web sites of companies that have conducted or were conducting RCTs on probiotics and prebiotics were searched.

Examples: Pfizer (http://www.pfizerpro.com/clinicaltrials), Chris Hansen Laboratory (http://www.chr-hansen.com/research_development/documentation.html). A search was conducted on prospective trial registries such as World Health Organization (WHO) International Clinical Trials Registry Platform Search Portal (http://www.who.int/trialsearch), Clinical Trials.gov register (http://www.clinicaltrials.gov), Current Controlled Trials *metaR*egister of Controlled Trials [*mRCT*] (http://www.controlled-trials.com/mrct) and http://www.clinicaltrialresults.org.

### Selection of studies

One reviewer (MM) independently reviewed all abstracts, citations and identified potentially eligible RCTs. The full reports of eligible RCTs were retrieved by one reviewer (MM) and the pre-specified selection criteria applied independently by two reviewers (MM, ML) using a study eligibility form designed for this review. If more than one publication of a study existed, all reports of the study were grouped together under one name. Any disagreements between the reviewers were resolved through discussion. Unresolved disagreements were resolved by a third party (RB).

### Data extraction and management

Two reviewers (MM, ML) independently extracted data using a pretested data extraction form that was designed for this review. The reviewers (MM, ML) cross checked data and resolved any differences through discussion. Unresolved disagreements were resolved by a third party (RB). One reviewer (MM) entered the data in SPSS version 19 and the other reviewer (AM) conducted quality control checks. The data obtained from each RCT included:

#### A) Source of funding or support of RCTs

The source of funding or support of the RCTs was defined and categorized as:

1) Industry included:

• For – profit company, donation of study product by a for – profit company which manufactured the study product,

• Not – for profit company that promoted the consumption of synbiotics, probiotics or prebiotics,

• Mixed sources (for-profit company and other source).

2) Non – industry included:

• Government: National, regional (provincial, county) government body with **NO** industry association.

• Foundation / Philanthropies: examples include Rockefeller foundation, Bill and Melinda Gates foundation.

• Institution: University, Research centres, teaching and academic hospitals.

• Other source of funding.

3) None: No source of funding was disclosed in study report.

#### B) Assessment of methodological quality of evidence (Risk of bias)

Two reviewers (MM, ML) independently assessed the risk of bias of included RCTs as described in the Cochrane Handbook for Systematic Reviews for Interventions according to the following 6 components: 1) sequence generation; 2) allocation concealment; 3) blinding; 4) incomplete outcome data; 5) selective outcome reporting; and 6) other sources of bias [17]. Each domain was assessed as having either a low risk of bias, high risk of bias or unclear to permit judgment. Any disagreements regarding risk of bias were resolved through discussion between MM, ML and RB. The association between risk of bias (domains) and type of funding (industry, non – industry, none declared) was explored.

#### C) Assessment of clinical outcomes

The primary and secondary outcomes from each study report were evaluated and categorized as:

1. Positive: synbiotic, probiotic or prebiotic supplementation had a statistically significant effect, p < 0.05.

Examples of positive outcomes included: adequate growth (weight gain, length gain, head circumference), tolerance (no feeding problems), microflora (increase in colony forming units of bifidobacteria, lactobacillus, decrease in pathogens), decreased infections (decrease in frequency, incidence of infections).

2) Negative: synbiotic, probiotic or prebiotic supplementation had a statistically significant effect *in an adverse event / negative outcome such as weight loss, diarrhoea*, p < 0.05

3) Neutral: synbiotic, probiotic or prebiotic supplementation did not have a statistically significant effect, p > 0.05, no significant differences between study groups. Clinical outcomes included: growth parameters, gastrointestinal parameters (tolerance to feed, stool characteristics, microflora); immune response, infections and mortality.

#### D) Overall study conclusions and conclusions on reported outcomes

The authors’ overall study conclusion and conclusions on reported clinical outcomes were evaluated and categorized as:

1. Positive: The author’s conclusion preferred the sponsor’s products over control/placebo. Interpretation of data supported the sponsor’s products over control.

2. Negative: The sponsors’ products were **not** preferred over control / placebo. Interpretation of data did NOT support the sponsors’ products.

3. Neutral: The author’s conclusion was neutral to the sponsor’s products.

4. No clear conclusion was offered by author.

In this review, the “conclusions on reported outcomes” referred to the authors’ conclusions on individual reported RCTs outcomes. Examples include conclusions on weight gain, length gain, vomiting, necrotizing enterocolitis, sepsis.

### Statistical Analysis

All the outcomes in this review were dichotomous and are described in frequencies and percentages. The association between source of funding (industry/non-industry/ none) and methodological quality (low/unclear/high risk of bias), clinical outcomes and author’s conclusions were assessed using both the Pearson’s Chi-square test and the Fisher’s exact test. A p-value of less than 0.05 was considered statistically significant. SPSS version 19 statistical software was used. A statistician (AM) was consulted throughout the review process.

### Ethics

The Human Research Ethics Committee at Stellenbosch University, South Africa reviewed the protocol for this review, ruled that all data to be collected for this review was from the public domain and was therefore exempt from ethical approval.

## Results

### Results of the search and description of studies

**Figure 1:**
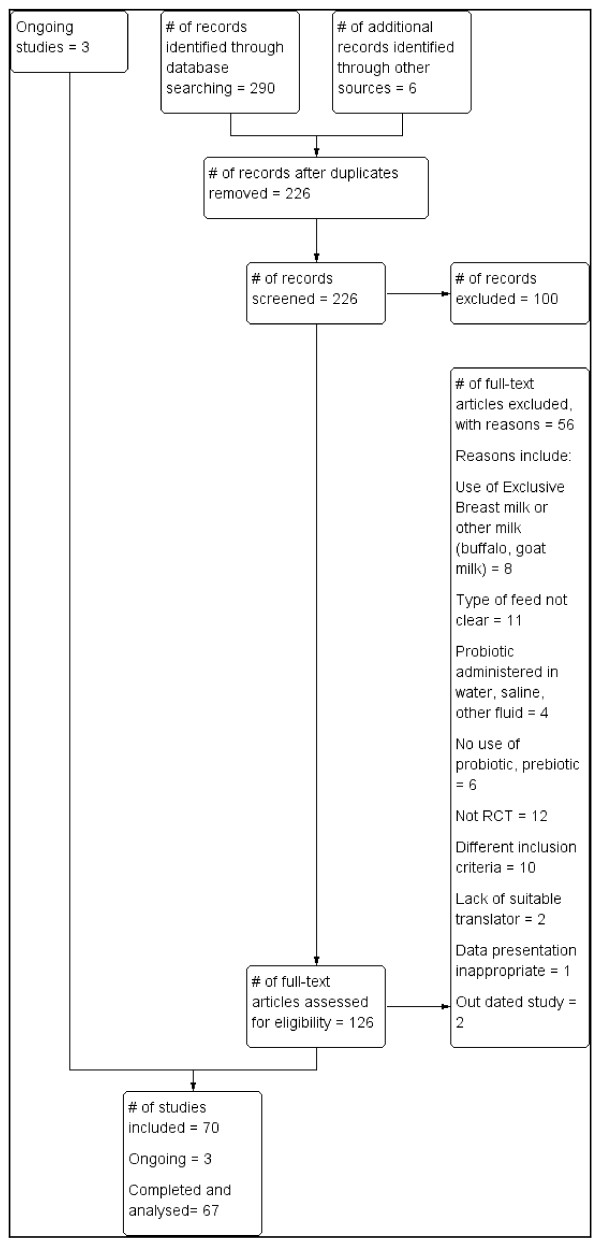
Process of study selection.

Electronic search of available databases yielded 290 citations. After reading titles and abstracts, duplicate reports were removed, 226 articles were screened and 100 articles were excluded. A hand search yielded 6 more articles. Potentially relevant full text reports were retrieved, reviewed for eligibility and a further 56 RCTs were excluded. Studies that had multiple publications were considered as one trial. Sixty seven RCTs and three on-going RCTs were included in this review. (Table 1) The selection process is shown in Figure 1. Table 2 gives a list of 56 RCTs which were excluded for: use of exclusive breast or non-formula milk (8 RCTs), type of feed not clear (11 RCTs), probiotic administered in saline, water or other fluid (4 RCTs), no use of probiotic or prebiotic (6 RCTs), not RCT (12 studies), different inclusion criteria (10 studies), lack of suitable translator (2 RCTs), data presentation inappropriate (1 RCT) and out of date [published before1980] (2 RCTs). Three excluded RCTs were unpublished trials.

**Table 1:** Included studies and on-going studies

**Included studies**						**On-going studies**	
**Author publication year**	**Full term/Preterm infant**	**Sponsor**	**Author publication year**	**Full term/Preterm infant**	**Sponsor**	**Author, Year study commenced**	**Full term/Preterm infant**
Allen 2010 [18]	Full Term	Knowledge exploitation fund, collaborative industrial research, others	Soh 2009 [19]	Full Term	National Medical Research Council, Singapore	Jacobs 2007 [20]	Pre-Term
Alliet 2007 [21] Scholtens 2008 [22]	Full Term	Numico	Urban 2008 [23]	Full Term	Nestle	Patole 2009 [24]	Pre-Term
Ashley 2012 [25]	Full Term	Mead Johnson	Velaphi 2008 [26]	Full Term	Nestle	Underwood 2009 [27]	Pre-Term
Bakker-Zierikzee 2005 [28] Bakker-Zierikzee 2006 [29]	Full Term	None/Not clear	Vendt 2006 [30]	Full Term	Valio Ltd		
Bettler 2006 [31]	Full Term	Wyeth Nutrition	Vlieger 2009 [32]	Full Term	Friesland		
Brunser 2006 [33]	Full Term	None/Not clear	Weizman 2005 [34]	Full Term	Materna Laboratories		
Bruzzese 2009 [35]	Full Term	Numico	Weizman 2006 [36]	Full Term	Marterna Laboratories		
Chouraqui 2004 [37]	Full Term	Nestle	Xiao-Ming 2004 [38]	Full Term	Friesland		
Chouraqui 2008 [39]	Full Term	Nestle	Xiao-Ming 2008 [40]	Full Term	None / Not clear		
Copper 2010 [41]	Full Term	Nestle	Ziegler 2007 [42]	Full Term	Mead Johnson		
Costalos 2008 [43]	Full Term	Numico	Bin-Nun 2005 [44]	Pre-Term	Mr and Mrs Stephen Hammerman, Mirsky Research fund		
Decsi 2005 [45]	Full Term	Numil Ltd	Boehm 2002 [46] Boehm 2003 [47] Knol 2005 [48]	Pre-Term	Numico		
Fanaro 2005 [49]	Full Term	None / Not clear	Chrzanowska-Liszewska 2012 [50]	Pre-Term	None/Not clear		
Fanaro 2008 [51]	Full Term	Humana GmbH	Costalos 2003 [52]	Pre-Term	None/Not clear		
Gibson 2009 [53]	Full Term	Nestle	Dani 2002 [54]	Pre-Term	None/Not clear		
Gil-Campos 2012 [55]	Full Term	Puleva	Indrio 2008 [56]	Pre-Term	Bio Gaia		
Hascoet 2011 [57]	Full Term	Nestle	Indrio 2009 [58]	Pre-Term	Numico		
Holscher 2012a [59]	Full Term	Nestle	Kapiki 2007 [60]	Pre-Term	None/Not clear		
Holscher 2012b [61]	Full Term	Nestle	Kitajima 1992 [62]	Pre-Term	None/Not clear		
Kim 2010 [63]	Full Term	Ministry of Health, Welfare and family affairs. Republic of Korea	Lin H-C 2008 [64]	Pre-Term	National Science Council of Taiwan		
Knol 2005 [65]	Full Term	Numico	Mihatsch 2006 [66]	Pre-Term	Milupa GmbH		
Magne 2008 [67]	Full Term	Numico	Mihatsch 2010 [68]	Pre-Term	Nestle		
Mah 2007 [69]	Full Term	National Medical Research Council Singapore	Millar 1993 [70] Stansbridge 1993 [71]	Pre-Term	Wessex Regional Health Authority and childrens Research fund		
Maldonado 2010 [72]	Full Term	Puleva	Modi 2010 [73]	Pre-Term	Danone		
Moro 2002 [74] Moro 2003 [75]	Full Term	None/Not clear	Mohan 2006 [76]	Pre-Term	None/Not clear		
Moro 2005 [77]	Full Term	None/Not clear	Reuman1986 [78]	Pre-Term	None/Not clear		
Moro 2006 [79]Arslanoglu 2007 [80] Arslanoglu 2008 [81] Van Hoffen 2009 [82] Schouten 2011 [83]	Full Term	Numico	Riskin 2009 [84]	Pre-Term	None/Not clear		
Piemontese 2011 [85]	Full Term	Danone	Rouge 2009 [86]	Pre-Term	French Ministry of Health		
Puccio 2007 [87]	Full Term	Nestle	Sari 2011 [88]	Pre-Term	None/Not clear		
Rautava 2006 [89] Rautava 2009 [90]	Full Term	Microbes and Man Research program, Academy of Finland, others	Stratiki 2007 [91]	Pre-Term	Nestle		
Rinne 2005 [92]	Full Term	Academy of Finland, Turku University Central Hospital Research Funds	Westerbeek 2010 [93] Westerbeek 2011a [94] Westerbeek 2011b [95]	Pre-Term	Danone		
Saavedra 2004 [96]	Full Term	Nestle	Yong 2009 [97]	Pre-Term	None/Not clear		
Scalabrin 2009 [98]	Full Term	Mead Johnson					
Scalabrin 2012 [99]	Full Term	Mead Johnson					
Schmelzle 2003 [100]	Full Term	Numico					

**Table 2:** Table of 56 Excluded studies with reasons for exclusion

**Use of Exclusive breast milk or Other milk feeds (buffalo, goat milk)**	**Type of feed not clear/specified**	**Probiotic administered in water, saline or other fluid that is not infant formula**	**No use of probiotic, prebiotic**	**Not RCT, (Cross over, Follow up, Observational study)**	**Different inclusion criteria**	**Lack of suitable/ knowledgeable translator**	**Data presentation inappropriate**	**Out dated (published before 1980)**
Agarwal 2003 [101]	Al Hosni 2012 [102]	FengJuan 2008 [103]	Morisset 2011 [104]	Huet 2006 [105]	Agustina 2007 [106]	Akiyama1994a [107] (Japanese)	Grzéskowak 2012 [108]	Andrews 1969 [109]
Baldeon 2008 [110]	Campeotto 2011 [111]	Kuitunen 2009 [112]	Patole 2005 [113]	Bongers 2007 [114]	Correa 2005 [115]	Akiyama1994b [116] (Japanese)		Robinson 1952 [117]
Braga 2011 [118]	Cukrowska 2002 [119]	Kukkonen 2007 [120]	Rochat 2007 [121]	Chou I-C 2009 [122]	Hol 2008 [123]			
Chandra 2002 [124]	*Karvonen 1999 [125]	Kukkonen 2008 [126]	Taipale 2011 [127]	Euler 2005 [128]	Isolauri 2000 [129]			
Lin H-C 2005 [130]	*Karvonen 2001 [131]		Taylor 2009 [132]	Hoyos 1999 [133]	Nopchinda 2002 [134]			
Manzoni 2006 [135]	*Karvonen 2002 [136]		Thibault 2004 [137]	Kim 2007 [138]	Rivero 2004 [139]			
Rinne 2006 [140]	Li 2004 [141]			Lee 2007 [142]	Urao 1999 [143]			
Samanta 2009 [144]	Panigrahi 2008 [145]			Lidesteri 2003 [146]	Van der Aa 2010 [147]			
	Rojas 2012 [148]			Marini 2003 [149]	Waliogora-Dupriet 2007 [150]			
	Taylor 2007 [151]			Rigo 2001 [152]	Wang 2007 [153]			
	Underwood 2009 [154]			Savino 2003 [155]				
				Sepp 1993 [156]				

Key: * Unpublished trials.

### Characteristics of included studies

Table 1 lists included and on-going trials. Sixty seven RCTs were included, 45 (67.2%) on full term infants, 22 (32.8%) on preterm infants. All included RCTs were published trials. All trials were conducted on healthy full term or preterm infants and used standard (full term or preterm) infant formula (Table 3).

**Table 3:** Source of funding and study participants

	**Study participants**		
**Sponsor**	**Full term infant**	**Preterm Infant**	**Total**
	**n**	**n**	**n (%)**
Industry	33	7	40 (59.7)
None / Not Clear	6	10	16 (23.9)
Non Industry	6	5	11 (16.4)
Total	45	22	67 (100.0)

### Funding

Out of 67 trials, 40 (59.7%) were funded by food industry, 11 (16.4%) were funded by non-industry entities, and 16 (23.9%) did not specify their source of funding, 10 RCTs on preterm infants, 6 RCTs on full infants (Table 3).

### Methodological quality (Risk of bias)

In this review, several domains were not adequately reported, particularly, the domains of sequence generation, allocation concealment and blinding. Out of 67 RCTs, 25 (37.3%) failed to report sequence generation, 35 (52.2%) failed to report allocation concealment and 36 (53.7%) did not report blinding. Majority of the RCTs were assessed as having a low risk of bias in the domains of incomplete outcome data 52 (77.6%), selective reporting 57(85.1%) and other bias 53 (79.1%) (Table 4).

**Table 4:** Methodological quality (Risk of bias)

	**N (%)**		
**Quality of studies N = 67**	**Low risk**	**High risk**	**Unclear**
Sequence generation	42 (62.7)		25 (37.3)
Allocation concealment	32 (47.8)		35 (52.2)
Blinding	31 (46.3)		36 (53.7)
Incomplete Outcome data	52 (77.6)	1 (1.5)	14 (20.9)
Selective reporting	57 (85.1)	7 (10.4)	3 (4.5)
Other bias	53 (79.1)		14 (20.9)

### Outcomes and study conclusions

In most RCTs, majority of outcomes were assessed as neutral, (intervention did not have a statistically significant effect, p > 0.05). A total of 49 (73.1%) of RCTs had a positive overall study conclusion in favour of the sponsors’ products, while 7 (10.4%) had negative¸ 7 (10.4%) had neutral conclusions and 4 (6%) had no clear conclusion. The included RCTs either did not provide any conclusion on their reported clinical outcomes or, they provided a positive conclusion for their reported outcome in-favour of the sponsors’ products. Few RCTS had either negative or neutral conclusions on their reported clinical outcomes (Table 5).

**Table 5:** Reported outcomes and conclusions

							**N (%)**			
					**Variable**	**N=**	**No conclusion**	**Positive**	**Neutral**	**Negative**
**Variable**		**N (%)**			**Overall study conclusion**	**67**	**4 (6)**	**49 (73.1)**	**7 (10.4)**	**7 (10.4)**
**Reported Outcomes**	**N=**	Positive*	Neutral*	Negative*	**Conclusion on reported outcomes**					
Weight gain	56	4 (7.1)	52 (92.9)		Weight gain	56	40 (71.4)	15 (26.8)		1 (1.8)
Length gain	40	3 (7.5)	37 (92.5)		Length gain	40	26 (65)	14 (35)		
Head circumference	31	4 (12.9)	27 (87.1)		Head circumference	31	17 (54.8)	14 (45.2)		
Colic	13	1 (7.7)	12 (92.3)		Colic	13	11 (84.6)	2 (15.4)		
Spitting up/Regurgitation	26	2 (7.7)	23 (88.5)	1 (3.8)	Spitting up/Regurgitation	26	23 (88.5)	3 (11.5)		
Vomiting	31	1.5 (3.2	30 (96.8)		Vomiting	32	24 (75)	8 (25)		
Crying/Fussiness	22	3 (13.6)	18 (81.8)	1 (4.5)	Crying/Fussiness	20	12 (60)	8 (40)		
Gastric Residuals, Abdominal distension	5	1 (20)	4 (80)		Gastric Residuals, Abdominal distension	6	3 (50)	3 (50)		
Volume of formula consumed	31	3 (9.7)	27 (87.1)	1 (3.2)	Volume of formula consumed	30	26 (86.7)	3 (10)		1(3.3)
Time to full enteral feeds	9	2 (22.2)	7 (77.8)		Time to full enteral feeds	8	5 (62.5)	2 (25)		1 (12.5)
Stool frequency	37	10 (27)	27 (73)		Stool frequency	38	27 (71.1)	11 (28.9)		
Stool consistency	37	18 (48.6)	19 (51.4)		Stool consistency	39	23 (59)	16 (41.0)		
Stool pH	13	11 (84.6)	2 (15.4)		Stool pH	12	7 (58.3)	5 (41.7)		
Short chain fatty acids	9	3 (33.3)	6 (66.7)		Short chain fatty acids	9	5 (55.6)	4 (44.4)		
Flatulence/Gas	16		16 (100)		Flatulence/Gas	15	11 (73.3)	4 (26.7)		
Diarrhoea, Diarrhoea episodes	19	3(15.8)	15 (78.9)	1(5.3)	Diarrhoea, Diarrhoea episodes	18	12 (66.7)	5 (27.8)		1 (5.6)
Constipation	3	1 (33.3)	2 (66.7)		Constipation	4	3 (75)	1 (25)		
Microflora - Bifidobacteria	31	23 (74.2)	8 (25.8)		Microflora - Bifidobacteria	30	10 (33.3)	17 (56.7)	2 (6.7)	1 (3.3)
Microflora - Lactobacillus	19	8 (42.1)	11 (57.9)		Microflora - Lactobacillus	19	9 (47.4)	8 (42.1)	1 (5.3)	1 (5.3)
Microflora - Pathogens	25	5 (20)	19 (76)	1 (4)	Microflora - Pathogens	25	12 (48)	11 (44)		2 (8)
Immune response CRP, IL6, Cytokines	0				Immune response CRP, IL6, Cytokines	1	1 (100)			
Immunoglobulins (IgA,IgG, Ig-Flc, IgE)	10	6 (60)	4 (40)		Immunoglobulins (IgA,IgG, Ig-Flc, IgE)	10	4 (40)	6 (60)		
Allergy	3	1 (33.3)	2 (66.7)		Allergy	3	2 (66.7)	1 (33.3)		
Eczema, Dermatitis, Rash, Skin Alterations	7	2 (28.6)	4 (57.1)	1 (14.3)	Eczema, Dermatitis, Rash, Skin Alterations	7	5 (71.4)	1 (14.3)		1 (14.3)
Infections - Acute Otitis Media	3		3 (100)		Infections - Acute Otitis Media	3	1 (33.3	2 (66.7)		
Respiratory Infections	9	3 (33.3)	6 (66.7)		Respiratory Infections	8	5 (62.5)	3 (37.5)		
Gastrointestinal infections	6	1 (16.7)	5 (83.3)		Gastrointestinal infections	4	1 (25)	3 (75)		
Total infections, other unspecified infections	8	1 (12.5)	7 (87.5)		Total infections, other unspecified infections	10	6 (60)	2 (20)		2 (20)
Urinary tract infections	2		2 (100)		Urinary tract infections	2	1 (50)	1 (50)		
Necrotizing Enterocolitis	11	2 (18.2)	9 (81.8)		Necrotizing Enterocolitis	12	7 (58.3)	3 (25)		2 (16.7)
Sepsis	10		10 (100)		Sepsis	10	9 (90)	1 (10)		
Fever, Febrile Episodes	4	2 (50)	2 (50)		Fever, Febrile Episodes	2	2 (100)			
Antibiotic use	19	4 (21.1)	15 (78.9)		Antibiotic use	16	13 (81.3)	3 (18.8)		
Hospitalization	12		12 (100)		Hospitalization	10	10 (100)			
Biochemical measures	9		9 (100)		Biochemical measures	6	5 (83.3)	1 (16.7)		
Adverse events	18	2 (11.1)	16 (88.9)		Adverse events	17	13 (76.5)	4 (23.5)		
Death / Mortality	7	1 (14.3	6 (85.7)		Death/Mortality	8	7 (87.5)	1 (12.5)		
Intestinal permeability	3	1 (33.3)	2 (66.7)		Intestinal permeability	3	1 (33.3)	2 (66.7)		
Duration of TPN	5		5 (100)		Duration of TPN	5	4 (80)	1 (20)		

*Positive: synbiotic, probiotic or prebiotic supplementation **had a** statistically significant effect, p < 0.05.

*Neutral: synbiotic, probiotic or prebiotic supplementation **did not have** a statistically significant effect, p > 0.05.

*Negative: synbiotic, probiotic or prebiotic supplementation **had a** statistically significant ***increase in an adverse event / negative outcome,*** p < 0.05.

### Association between source of funding (sponsor) and methodological quality of studies

There was no significant association between the source of funding and the domains of sequence generation (Chi – square p = 0.435, Fisher exact p = 0.465), allocation concealment (Chi – square p = 0.315, Fisher exact p = 0.338), blinding (Chi – square p = 0.395, Fisher exact p = 0.457) and selective reporting (Chi – square p = 0.224, Fisher exact p = 0.188) (Table 6).

**Table 6:** Association between Sponsor and methodological quality (risk of bias)

**Methodological quality**	**Source of funding**	**Yes (Low risk)**	**No (High risk)**	**Unclear**	**Chi-square p value**	**Fisher’s exact p value**
**N = 67 studies**		**n (%)**^**$$**^	**n (%)**^**$$**^	**n (%)**^**$$**^		
**Sequence generation**	Industry	26 (38.8)		14 (20.9)	0.435	0.465
	None/Not clear	8 (11.9)		8 (11.9)		
	Non industry	8 (11.9)		3 (4.5)		
**Allocation concealment**	Industry	21 (31.3)		19 (28.4)	0.315	0.338
	None/Not clear	5 (7.5)		11 (16.4)		
	Non Iindustry	6 (9.0)		5 (7.5)		
**Blinding**	Industry	18 (26.9)		22 (32.8)	0.395	0.457
	None/Not clear	6 (9.0)		10 (14.9)		
	Non industry	7 (10.4)		4 (6.0)		
**Incomplete outcome data**	Industry	36 (53.7)	1 (1.5)	3 (4.5)	0.023*	0.005*
	None/Not clear	9 (13.4)		7 (10.4)		
	Non industry	7 (10.4)		4 (6.0)		
**Selective reporting**	Industry	36 (53.7)	2 (3.0)	2 (3.0)	0.224	0.188
	None/Not clear	11 (16.4)	4 (6.0)	1 (1.5)		
	Non industry	10 (14.9)	1 (1.5)	0		
**Free of other bias**	Industry	35 (52.2)		5 (7.5)	0.033*	0.038*
	None/Not clear	9 (13.4)		7 (10.4)		
	Non industry	8 (13.4)	1 (1.5)	2 (3.0)		

*Significant p < 0.05.

^$$^Overall percentage.

There was a significant association between funding and the domains of incomplete outcome data (Chi – square p = 0.023, Fisher exact p = 0.005) and free of other bias (Chi – square p = 0.033, Fisher exact p = 0.038) (Table 6). The association between source of funding and incomplete outcome data was such that industry-funded trials had significantly less missing data than non-industry funded trials. The association between source of funding and free of other bias (such as outcomes bias) was such that a significantly higher percentage of industry-funded trials were free of other bias compared to non-industry-funded trials.

### Association between source of funding (sponsor) and clinical outcomes

There was no significant association between source of funding and reporting of clinical outcomes: Growth parameters, stool characteristics, microflora, infections (Tables 7, 8, 9, 10 and 11), immune parameters, adverse events and mortality (data not shown). There was a significant association between the source of funding and reporting of antibiotic use in formula fed infants (Chi-square p = 0.031, Fisher exact p = 0.039) such that industry funded trials were more likely to decrease the use of antibiotics than non-industry funded trials (Table 11).

**Table 7:** Association between Sponsor and clinical outcomes: Growth

		**Assessment of outcome**			
**Growth**	**Source of funding**	Positive*	Neutral*	**Chi-square p value**	**Fisher’s exact p value**
		**n (%)**^**$$**^	**n (%)**^**$$**^		
Weight gain N = 56	Industry	2 (3.6)	35 (62.5)	0.309	0.266
	None/Not clear	2 (3.6)	10 (17.9)		
	Non industry	0	7 (12.5)		
Length gain N = 40	Industry	3 (7.5)	29 (72.5)	0.667	1.00
	None/Not clear		6 (15)		
	Non industry		2 (5)		
Head Circumference N = 31	Industry	4 (12.9)	23 (74.2)	0.712	1.00
	None /Not clear		3 (9.7)		
	Non industry		1 (3.2)		

^$$^Overall percentage.

*Positive: synbiotic, probiotic or prebiotic supplementation **had a** statistically significant effect, p < 0.05. **There were significant differences** between study groups **(in favour of experimental group)**.

*Neutral: synbiotic, probiotic or prebiotic supplementation **did not have** a statistically significant effect, p > 0.05, **No significant differences** between study groups.

**Table 8:** Association between Sponsor and clinical outcomes: Tolerance symptoms

**Tolerance**	**Source of funding**	Positive*	Negative*	Neutral*	**Chi-square p value**	**Fisher’s exact p value**
		**n (%)**^**$$**^	**n (%)**^**$$**^	**n (%)**^**$$**^		
Colic N = 13	Industry	1 (7.7)		11 (84.6)	0.764	1.00
	None/Not clear					
	Non industry			1 (7.7)		
Spitting up/Regurgitation N = 26	Industry	2 (7.7)	1 (3.8)	17 (65.4)	0.907	1.00
	None/Not clear			4 (15.4)		
	Non industry			2 (7.7)		
Vomiting N = 31	Industry	1 (3.2)		23 (74.2)	0.860	1.00
	None/Not clear			5 (16.1)		
	Non industry			2 (6.5)		
Crying fussiness N =22	Industry	3 (13.6)	1 (4.5)	14 (63.6)	0.581	1.00
	None/Not clear			4 (18.2)		
	Non industry			0		
Gastric residuals, Abdominal distension N = 5	Industry			1 (20)	0.659	1.00
	None/Not clear			1 (20)		
	Non industry	1 (6.7)		2 (40)		
Volume of formula consumed/daily intake N = 31	Industry	3 (9.7)	1 (3.2)	18 (58.1)	0.758	1.00
	None/Not clear			4 (12.9)		
	Non industry			5 (16.1)		
Days to full enteral feeding N = 9	Industry			4 (44.4)	0.325	0.444
	None/Not clear	1 (11.1)		1 (11.1)		
	Non industry	1 (11.1)		2 (22.2)		

^**$$**^Overall percentage.

*Positive: synbiotic, probiotic or prebiotic supplementation **had a** statistically significant effect, p < 0.05. **There were significant differences** between study groups **(in favour of experimental group)**.

*Neutral: synbiotic, probiotic or prebiotic supplementation **did not have** a statistically significant effect, p > 0.05, **No significant differences** between study groups.

*Negative: synbiotic, probiotic or prebiotic supplementation **had a** statistically significant ***increase in an adverse event / negative outcome,*** p < 0.05.

**Table 9:** Association between sponsor and clinical outcomes: stool characteristics

**Stool characteristics**	**Source of funding**	Positive*	Negative*	Neutral*	**Chi-square p value**	**Fisher’s exact p value**
		**n (%)**^**$$**^	**n (%)**^**$$**^	**n (%)**^**$$**^		
Stool Frequency N = 37	Industry	7 (18.9)		22 (59.5)	0.501	0.540
	None/Not clear	3 (8.1)		4 (10.8)		
	Non industry			1 (2.7)		
Stool Consistency n =37	Industry	14 (37.8)		15 (40.5)	0.562	1.00
	None/Not clear	4 (10.8)		3 (8.1)		
	Non industry			1 (2.7)		
Stool pH N =13	Industry	7 (53.8)		2 (15.4)	0.305	1.00
	None/Not clear	4 (30.8)				
	Non industry					
Stool Short Chain Fatty Acids N = 9	Industry	2 (22.2)		4 (44.4)	0.687	1.00
	None / Not clear	1 (11.1)		1 (11.1)		
	Non industry			1 (11.1)		
Flatulence / Gas N = 16	Industry			15 (93.8)	Not valid	
	None/Not clear			1 (6.3)		
	Non industry			0		
Diarrhoea, Diarrhoea episodes N = 19	Industry	3 (15.8)	1 (5.3)	10 (52.6)	0.771	1.00
	None/Not clear			2 (10.5)		
	Non industry			3 (15.8)		
Constipation N = 3	Industry	1 (33.3)		1 (33.3)	0.386	1.00
	None/Not clear			1 (33.3)		
	Non industry			0		

^**$$**^Overall percentage.

*Positive: synbiotic, probiotic or prebiotic supplementation had a statistically significant effect, p < 0.05. There were significant differences between study groups (in favour of experimental group).

*Neutral: synbiotic, probiotic or prebiotic supplementation did not have a statistically significant effect, p > 0.05, No significant differences between study groups.

*Negative: synbiotic, probiotic or prebiotic supplementation had a statistically significant increase *in an adverse event / negative outcome,* p < 0.05.

**Table 10:** Association between sponsor and clinical outcomes: Microflora

**Microflora**	**Source of funding**	Positive 4*	Negative 5*	Neutral 6*	**Chi-square p value**	**Fisher’s exact p value**
		**n (%)**^**$$**^	**n (%)**^**$$**^	**n (%)**^**$$**^		
Bifidobacteria N = 31	Industry	12 (38.7)		6 (19.4)	0.416	0.583
	None/Not clear	8 (25.8)		2 (6.5)		
	Non industry	3 (9.7)				
Lactobacillus N = 19	Industry	2 (10.5)		6 (31.6)	0.155	0.176
	None/Not clear	4 (21.1)		5 (26.3)		
	Non industry	2 (10.5)		0		
Pathogens N = 25	Industry	2 (8.0)		11 (44.0)	0.532	0.612
	None/Not clear	3 (12.0)	1 (4.0)	6 (24.0)		
	Non industry			2 (8.0)		

^**$$**^ Overall percentage.

*Positive: synbiotic, probiotic or prebiotic supplementation **had a** statistically significant effect, p < 0.05. **There were significant differences** between study groups **(in favour of experimental group)**.

*Neutral: synbiotic, probiotic or prebiotic supplementation **did not have** a statistically significant effect, p > 0.05, **No significant differences** between study groups.

*Negative: synbiotic, probiotic or prebiotic supplementation **had a** statistically significant ***increase in an adverse event / negative outcome,*** p < 0.05.

**Table 11:** Association between sponsor and clinical outcomes: Necrotizing enterocolitis, sepsis and antibiotic use

	**Source of funding**	Positive*	Neutral*	**Chi-square p value**	**Fisher’s exact p value**
		**n (%)**^**$$**^	**n (%)**^**$$**^		
Necrotising enterocolitis N = 11	Industry		4 (36.4)	0.118	0.273
	None/Not clear		3 (27.3)		
	Non industry	2 (18.2)	2 (18.2)		
Sepsis N = 10	Industry		2 (20)	Not Valid	
	None/Not clear		3 (30)		
	Non industry		5 (50)		
Antibiotic use N = 19	Industry	4 (21.1)	4 (21.1)	0.031^#^	0.039^#^
	None/Not clear		5 (26.3)		
	Non industry		6 (31.6)		

^$$^ Overall percentage.

*Positive: synbiotic, probiotic or prebiotic supplementation **had a** statistically significant effect, p < 0.05. **There were significant differences** between study groups **(in favour of experimental group)**.

*Neutral: synbiotic, probiotic or prebiotic supplementation **did not have** a statistically significant effect, p > 0.05, **No significant differences** between study groups.

^#^ Significant p < 0.05.

### Association between source of funding (sponsor) and overall study conclusion

There was no significant association between sources of funding and overall study conclusion (Chi-square p = 0.505, Fisher exact p = 0.373). Majority of RCTs, 49 (73.1%), had a positive study conclusion; 32 (47.8%) of these RCTs, were industry sponsored, 7 (10.4%) non- industry and 10 (14.9%) which did not declare their source of funding (Table 12). A sensitivity analysis was conducted with respect to combining industry sponsored studies with those that had not declared their source of funding. There was no change in the results. There was no significant association between source of funding and overall study conclusion (Chi-square p = 0.483, Fisher exact p = 0.425).

**Table 12:** Association between sponsor and OVERALL study conclusion

	**Source of funding**	**Positive**	**Negative**	**Neutral**	**No clear conclusion**	**Total**	**Chi-square p value**	**Fisher’s exact p value**
		**n (%)**^**$$**^	**n (%)**^**$$**^	**n (%)**^**$$**^	**n (%)**^**$$**^	**n (%)**^**$$**^		
Overall conclusion N = 67	Industry	32 (47.8)	2 (3.0)	3 (4.5)	3 (4.5)	40 (59.7%)	0.505	0.373
	None/Not clear	10 (14.9)	3 (4.5)	2 (3.0)	1 (1.5)	16 (23.9%)		
	Non industry	7 (10.4)	2 (3.0)	2 (3.0)	0	11 (16.4%)		
	Total	49 (73.1%)	7 (10.4%)	7 (10.4%)	4 (6.0%)	67 (100)		

^**$$**^ Overall percentage.

### Association between source of funding (sponsor) and conclusion on reported clinical outcomes

There was a significant association between source of funding and conclusion on weight gain (Chi-square p = 0.037, Fisher exact p = 0.024) such that industry-funded trials were more likely to report positive conclusions on weight gain than non-industry-funded trials (Table 13). There was no significant association between source of funding and conclusion on other reported clinical outcomes (Tables 14, 15, 16 and 17).

**Table 13:** Association between sponsor and conclusion on reported outcome: Growth parameters

**Authors conclusion on:**	**Source of funding**	**No conclusion on reported outcome**	**Positive**	**Negative**	**Chi-square p value**	**Fisher’s exact p value**
		**n (%)**^**$$**^	**n (%)**^**$$**^	**n (%)**^**$$**^		
Weight gain N = 56	Industry	23 (41.1%)	14 (25.0%)		0.037^#^	0.024^#^
	None/Not clear	10 (17.9%)	1 (1.8%)	1 (1.8%)		
	Non industry	7 (12.5%)				
Length gain N = 40	Industry	18 (45%)	14 (35%)		0.068	0.051
	None/Not clear	6 (15%)				
	Non industry	2 (5)				
Head circumference N = 31	Industry	13 (41.9)	14 (45.2)		0.151	0.232
	None/Not clear	3 (9.7)				
	Non industry	1 (3.2)				

^#^Significant p < 0.05, ^**$$**^ Overall percentage.

**Table 14:** Association between sponsor and conclusion on reported outcome: Tolerance symptoms

**Tolerance**	**Source of funding**	**No conclusion on reported outcome**	**Positive**	**Negative**	**Chi-square p value**	**Fisher’s exact p value**
		**n (%)**^**$$**^	**n (%)**^**$$**^	**n (%)**^**$$**^		
Colic N = 13	Industry	10 (76.9)	2 (15.4)		0.657	1.00
	None/Not clear					
	Non industry	1 (7.7)				
Spitting up/Regurgitation N = 26	Industry	19 (73.1)	1 (3.8)		0.032	0.062
	None/Not clear	2 (7.7)	2 (7.7)			
	Non industry	2 (7.7)				
Vomiting N = 32	Industry	19 (59.4)	5 (15.6)			
	None/Not clear	3 (9.4)	3 (9.4)			
	Non industry	2 (6.3)				
Crying Fussiness N =20	Industry	10 (50)	6 (30)		0.648	1.00
	None/Not clear	2 (10)	2 (10)			
	Non industry	0				
Gastric residuals, Abdominal distension N = 6	Industry		1 (16.7)		0.513	1.00
	None/Not clear	1 (16.7)	1 (16.7)			
	Non industry	2 (33.3)	1 (16.7)			
Volume of formula consumed/daily intake N = 30	Industry	19 (63.3)	2 (6.7)	1 (3.3)	0.867	0.733
	None/Not clear	3 (10.0)				
	Non industry	4 (13.3)	1 (3.3)			
Days to full enteral feeding N = 8	Industry	2 (25)		1 (12.5)	0.547	1.00
	None/Not clear	1 (12.5)	1 (12.5)			
	Non industry	2 ()25	1 (12.5)			

^$$^Overall percentage.

**Table 15:** Association between sponsor and conclusion on reported outcome: Stool characteristics

**Stool characteristics**	**Source of funding**	**No conclusion on reported outcome**	**Positive**	**Negative**	**Pearson’s chi Square**	**Fisher’s exact p value**
		**n (%)**^**$$**^	**n (%)**^**$$**^	**n (%)**^**$$**^		
Stool frequency N = 38	Industry	21 (55.3)	9 (23.7)		0.809	1.00
	None / Not clear	5 (13.2)	2 (5.3)			
	Non industry	1 (2.6)				
	Total	27 (71.1)	11 (28.9)			
Stool consistency n =39	Industry	18 (46.2)	13 (33.3)		0.699	1.00
	None / Not clear	4 (10.3)	3 (7.7)			
	Non industry	1 (2.6)				
	Total	23 (59)	16 (41)			
Stool pH N =12	Industry	5 (41.7)	3 (25)		0.679	1.00
	None / Not clear	2 (16.7)	2 (16.7)			
	Non industry					
	Total	7 (58.3)	5 (41.7)			
Stool short chain fatty acids N = 9	Industry	3 (33.3)	3 (33.3)		0.638	1.00
	None / Not clear	1 (11.1)	1 (11.1)			
	Non industry	1 (11.1)				
	Total	5 (55.6)	4 (44.4)			
Flatulence/Gas N = 15	Industry	10 (66.7)	4 (26.7)		0.533	1.00
	None / Not clear					
	Non industry	1 (6.7)				
	Total	11 (73.3)	4 (26.7)			
Diarrhoea, Diarrhoea episodes N = 18	Industry	7 (38.9)	5 (27.8)	1 (5.6)	0.484	0.557
	None / Not clear	2 (11.1)				
	Non industry	3 (16.7)				
	Total	12 (66.7)	5 (27.8)	1 (5.6)		
Constipation N = 4	Industry	2 (50)	1 (25)		0.505	1.00
	None / Not clear					
	Non industry	1(25)				
	Total	3 (75)	1 (25)	1 (25)		

^**$$**^ Overall percentage.

**Table 16:** Association between sponsor and conclusion on reported outcome: Microflora

**Microflora**	**Source of funding**	**No conclusion on reported outcome**	**Positive**	**Negative**	**Neutral**	**Chi-square p value**	**Fisher’s exact p value**
		**n (%)**^**$$**^	**n (%)**^**$$**^	**n (%)**^**$$**^	**n (%)**^**$$**^		
Bifidobacteria N = 30	Industry	7 (23.3)	11 (36.7)			0.249	0.195
	None/Not clear	2 (6.7)	5 (16.7)	1 (3.3)	1 (3.3)		
	Non industry	1 (3.3)	1 (3.3)		1 (3.3)		
Lactobacillus N = 19	Industry	5 (26.3)	4 (21.1)			0.084	0.294
	None/Not clear	3 (15.8)	4 (21.1)		1 (5.3)		
	Non industry	1 (5.3)		1 (5.3)			
Pathogens N = 25	Industry	7 (28)	6 (24)			0.152	0.269
	None/Not clear	4 (16)	5 (20)	1 (4)			
	Non industry	1 (4)		1 (4)			

^$$^Overall percentage.

**Table 17:** Association between sponsor and conclusion on reported outcome: Necrotising Enterocolitis Sepsis and antibiotic use

	**Source of funding**	**No conclusion on reported outcome**	**Positive**	**Negative**	**Pearson’s chi Square**	**Fisher’s exact p value**
		**n (%)**^**$$**^	**n (%)**^**$$**^	**n (%)**^**$$**^		
NEC N = 12	Industry	3 (25)		1 (8.3)	0.511	0.782
	None/Not clear	2 (16.7)	1 (8.3)	1 (8.3)		
	Non industry	2 (16.7)	2 (16.7)	0		
		7 (58.3)	3 (25)	2 (16.7)		
Sepsis N = 10	Industry	2 (20)			0.274	0.500
	None/Not clear	2 (20)		1 (10)		
	Non industry	5 (50)				
Antibiotic use N = 16	Industry	4 (25)	3 (18.8)		0.093	0.141
	None/Not clear	4 (25)				
	Non industry	5 (31.3)				

^**$$**^ Overall percentage.

## Discussion

This review revealed that majority of RCTs (from 1980 to 2012) on infants fed formula supplemented with probiotics, prebiotics or synbiotics are funded by the food industry. This is consistent with the trend that biomedical research is increasingly being funded by industry [1,2] There was a trend that more RCTs on preterm infants failed to report their source of funding. The reason(s) for this trend needs to be explored further.

Cochrane guidelines were used to assess the risk of bias of included RCTs. The reporting of several domains was however suboptimal particularly sequence generation, allocation concealment and blinding domains. Considering completed data, there was no significant association between funding source and methodological quality of RCTs in the domains of sequence generation, allocation concealment, blinding and selective reporting. There was a significant association between funding and methodological quality of RCTs in the domains of incomplete outcome data and free of other bias. Industry funded trials had significantly less missing data than non-industry funded trials. A higher percentage of industry funded trials were free of other bias compared to non-industry funded trials. More industry sponsored trials had low risk of bias in 5 out of 6 domains, even though our results did not show a statistical significant association between funding and methodological quality in most domains. Our results confirm findings from previous reviews on infants given enteral feeds with probiotics, prebiotics and synbiotics [157-159].

There was no significant association between funding source and clinical outcomes or majority of authors’ conclusions. There was a significant association between funding and conclusion on weight gain. Regardless of the reported clinical outcomes, nearly all RCTs in this review reported neutral results. That is supplementation with probiotics, prebiotics or synbiotics did not have a significant effect or there were no significant differences between study groups of infants given supplemented formula or placebo. Our findings confirm the results of two systematic reviews which found that supplementation with probiotics, prebiotics or synbiotics did not offer any distinct advantage over placebo [158,159]. However, results of this review did not agree with two nutrition related reviews or reviews on pharmaceutical industry supported RCTs, which reported that industry sponsored RCTs had results and conclusions in favour of the sponsor [2-4,6,8,160-162]. Despite reporting neutral outcomes, authors from industry sponsored RCTs had a tendency to advocate for the consumption of the sponsors’ products. Similar findings were reported by Nestle, who reported that research investigators “who received company grants tended to publish results, give advice and prescribe in favour of the sponsor.” This applied to research that was supported by pharmaceutical and food industries [163].

Effects of sponsorship on overall study conclusion have been equally documented in biomedical literature. Reviews by Lessor and Nkansah reported positive conclusions in favour of the sponsor [6,8]. Although no statistically significant association between funding and authors conclusion was found in this review, more than 70% of RCTs reported positive conclusions, 47.8% of these were industry sponsored. Often, these positive conclusions in the RCTs were not supported by the reported data as demonstrated by the neutral clinical outcomes. Our findings are consistent with those of previous reviews, which found that, results from RCTs may be accurate, but authors may distort the meaning of the results, present conclusions that are more favourable, and that were not supported by the data presented [2,5,163]. Even meta – analyses were not spared from this trend [2,5,163]. Despite overwhelming positive overall study conclusions, majority of RCTs did not have any conclusion on their reported clinical outcomes. The RCTs that reported any conclusion on their clinical outcomes, majority were positive in favour of the sponsors’ products.

### Limitations

This review did not document the role of the sponsor in study design, data collection, and analysis. Few RCTs reported this. More detailed documentation and disclosure in RCT reports would help evaluate if there was an association between funding and reported outcomes or conclusions. Many RCTs had missing data especially on the domains of sequence generation, allocation concealment and blinding. Attempts were made to contact authors for missing information but none responded. The sample size (number of RCTs) was small and skewed towards industry.

## Conclusion

This study assessed the impact of funding by the food industry on trial outcomes and methodological quality of synbiotics, probiotics and prebiotics research in infants. There was no significant association between source of funding and methodological quality of study in the domains of sequence generation, allocation concealment and blinding. Industry funded trials had less missing data and were free of other bias than non-industry funded trials.

There was no significant association between funding and majority of reported clinical outcomes or authors’ conclusions. However, there was a significant association between funding source and reported antibiotic use and conclusion on weight gain. Majority of RCTs were industry funded, more non-industry funded research is needed to further assess the impact of funding on methodological quality, reported clinical outcomes and authors’ conclusions.

## Competing interests

The authors declare that they have no competing interests.

## Authors’ contributions

The reviewers contributed the following: MM: Developed review protocol (unpublished), selected RCTs, conducted data extraction, assessment of risk of bias in included RCTs, developed, edited and critically reviewed the manuscript. ML: Selected RCTs, conducted data extraction, assessment of risk of bias in included RCTs, critically reviewed the manuscript. AM: Conducted the statistical analysis, interpretation of results and critically reviewed the manuscript. TY: Contributed to designing the review methodology and critically reviewed the manuscript. RB: Contributed to designing the review, acted as third party arbitrator and critically reviewed the manuscript. All authors read and approved the final manuscript.

## Pre-publication history

The pre-publication history for this paper can be accessed here:

http://www.biomedcentral.com/1471-2288/13/137/prepub
